# Novel *NARS2* variant causing leigh syndrome with normal lactate levels

**DOI:** 10.1038/s41439-022-00191-z

**Published:** 2022-05-04

**Authors:** Ryosuke Tanaka, Ryo Takeguchi, Mami Kuroda, Nao Suzuki, Yoshio Makita, Kumiko Yanagi, Tadashi Kaname, Satoru Takahashi

**Affiliations:** 1 Department of Pediatrics, Asahikawa Medical University, Asahikawa, Japan; 2 Department of Genetic Counseling, Asahikawa Medical University Hospital, Asahikawa, Japan; 3 Department of Genome Medicine, National Center for Child Health and Development, Tokyo, Japan

**Keywords:** Paediatric neurological disorders, Neurodegeneration

## Abstract

Leigh syndrome is the most genetically heterogenous phenotype of mitochondrial disease. We describe a patient with Leigh syndrome whose diagnosis had not been confirmed because of normal metabolic screening results at the initial presentation. Whole-exome sequencing identified pathogenic variants in *NARS2*, the gene encoding a mitochondrial asparaginyl-tRNA synthetase. One of the biallelic variants was novel. This highlights the essential role of genetic testing for a definite diagnosis of Leigh syndrome.

Leigh syndrome (MIM 516060) is the most common childhood mitochondrial disorder and has an estimated prevalence of 1 per 40,000 live births^1^. This neurodegenerative disorder is genetically heterogeneous, and approximately 100 causative genes have been identified in either the mitochondrial or the nuclear genomes to date^2^. Increased lactate is an important biochemical marker in the diagnosis of patients with suspicion of mitochondrial disorders. Here, we report a case of a 24-year-old female with Leigh syndrome whose diagnosis had not been confirmed because she had normal blood and cerebrospinal fluid (CSF) lactate concentrations at presentation. Whole-exome sequencing (WES) analysis identified biallelic pathogenic variants in *NARS2* (MIM 612803). The gene is located on 11q14.1 and encodes mitochondrial asparaginyl-tRNA synthetase 2. The enzyme catalyzes the ligation of asparagine to tRNA molecules, so it plays a critical role in protein biosynthesis^3^. To date, 18 different *NARS2* disease-causing variants have been described in 22 affected patients^4–13^. This report aimed to better understand the phenotypic variability of *NARS2*-associated disease and the potential diagnostic pitfall.

The patient, now aged 24 years, was the first child of healthy, nonconsanguineous Japanese parents, and her sister was healthy. The patient was born at 40 weeks gestation after an uneventful pregnancy. Her birth weight, length, and head circumference were 2700 g (−0.9 SD), 48 cm (−0.8 SD), and 30 cm (−2.6 SD), respectively. No signs of perinatal distress were observed. Her psychomotor development was normal until one year of age; she acquired head control, sat without support, and stood with assistance by three months, seven months, and by 12 months of age, respectively. She suffered from a urinary tract infection at one year and one month of age and developed generalized tonic and clonic seizures. Thereafter, she exhibited developmental regression, which she lost her ability to stand and maintain a sitting position by one year and five months. Brain magnetic resonance imaging (MRI) at one year and seven months demonstrated symmetrical faint high signal lesions in the putamen (Fig. 1a). The patient developed generalized tonic and myoclonic seizures at one year and eight months of age. Electroencephalography showed generalized spike-waves. The antiepileptic drug valproic acid (VPA) was administered, but she had a cluster of tonic seizures. At the age of two years and three months, she was referred to our hospital. Neurological examination revealed muscle hypotonia and a profound delay in psychomotor development. She also showed involuntary movements, such as chorea and intermittent opisthotonos posturing. The auditory brainstem response test revealed severe bilateral auditory impairment. Laboratory examinations revealed normal lactate levels in both the blood and CSF (12 mg/dl and 10 mg/dl, respectively). Follow-up MRIs revealed symmetrical high signal intensity lesions involving the putamen and periaqueductal gray matter, which are characteristic of Leigh syndrome (Fig. 1). However, single-voxel proton magnetic resonance spectroscopy (MRS) obtained from the abnormal regions in the basal ganglia did not show a lactate peak (Supplementary Fig. 1). At the age of three years, she developed VPA-induced Fanconi syndrome that was detected incidentally with findings such as hypophosphatemia, metabolic acidosis with a normal anion gap, glycosuria, and generalized hyperaminoaciduria. However, these findings were normalized after VPA administration was stopped. During infections, the patient was repeatedly found to have liver dysfunction (aspartate aminotransferase, up to 469 U/L; and alanine aminotransferase, up to 278 U/L), which was coincident with increased lactate and pyruvate levels in blood (lactate, up to 46.0 mg/dl; pyruvate, up to 2.1 mg/dl). In the following years, the patient exhibited a slowly progressive clinical course. Her psychomotor development was severely delayed without head control, eye pursuit, or the use of meaningful words. Because of repeated aspiration pneumonia owing to feeding and swallowing difficulties, she was fed via a gastrostomy tube and required tracheostomy tube placement.

**Fig. 1:**
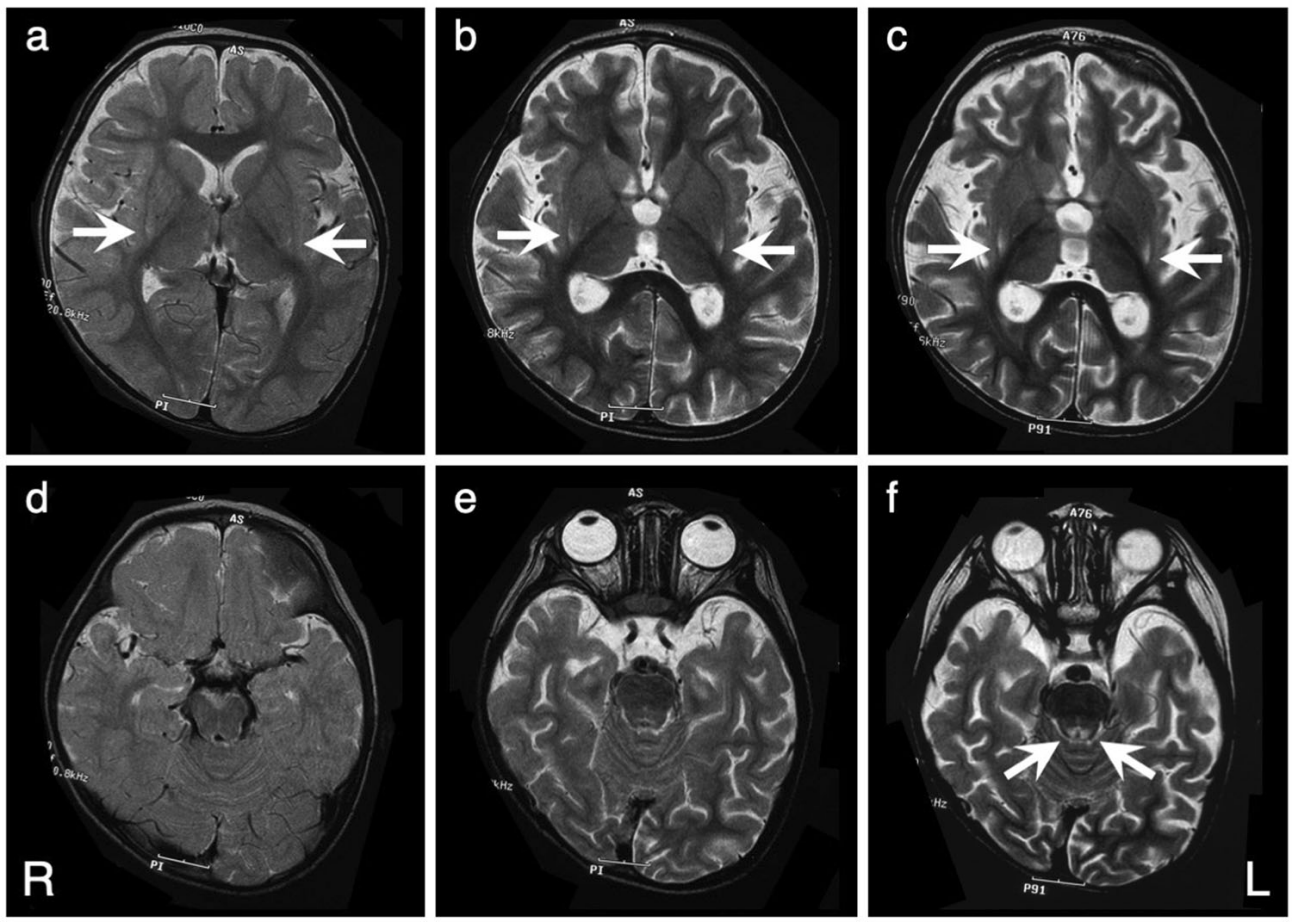
Serial changes in brain T2-weighted MRI findings in a patient with compound heterozygous variants in *NARS2*. Note the bilateral symmetric high signal lesions involving the dorsal putamen (arrows, **a**–**c**) and diffuse progressive cerebral atrophy at the age of 1.7 (**a**, **d**), 2.3 (**b**, **e**), and 3.3 (**c**, **f**) years old. Brain stem lesions become apparent at the age of 3.3 years (arrows, **f**).

To reveal the underlying genetic etiology of Leigh syndrome, WES analysis was conducted for the patient and her asymptomatic parents after written informed consent was obtained from her parents. The study protocol was approved by the Committee for Ethical Issues at Asahikawa Medical University (approval number 17112). WES analysis revealed that the patient harbored compound heterozygous variants in *NARS2*, with a paternally inherited NM_024678:c.731 C > G (p.Ala244Gly) variant and a maternally inherited NM_024678:c.556 A > G (p.Asn186Asp) variant. These variants were confirmed by Sanger sequencing (Fig. 2a). Both variants occurred at the catalytic domain of NARS2 (Fig. 2b, c). The paternal variant, p.Ala244Gly, has been reported in a patient with Leigh syndrome^8^. This variant was predicted to be “probably damaging” with a score of 0.983 by PolyPhen-2 and “deleterious” with a score of 0.001 by SIFT. The maternal variant, p.Asn186Asp, was not registered in the Genome Aggregation Database (http://gnomad.broadinstitute.org) or Human Genome Mutation Database (http://www.hgmd.cf.ac.uk/). The variant was predicted to be “probably damaging” with a score of 0.999 by PolyPhen-2 and “deleterious” with a score of 0.001 by SIFT. Based on the American College of Medical Genetics and Genomics standards and guidelines, these variants were classified as likely pathogenic.

**Fig. 2:**
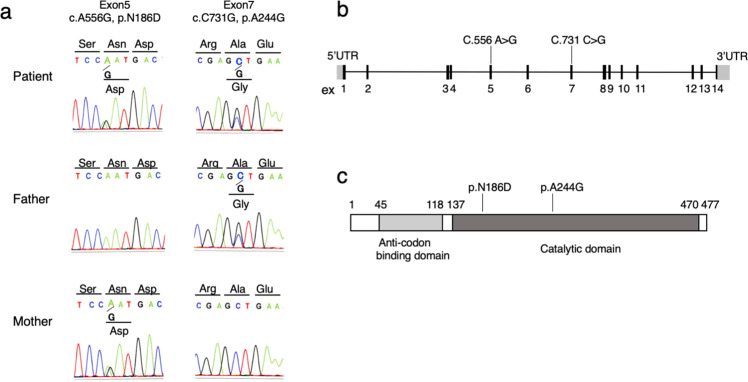
Genetic analysis of the patient. **a** Partial sequence chromatograms for *NARS2* in the patient and her parents. The patient harbors the biallelic heterozygous variants, with a paternally inherited NM_024678:c.731 C > G (p.Ala244Gly) variant and a maternally inherited NM_024678:c.556 A > G (p.Asn186Asp) variant. **b** Schematic illustration of *NARS2* at the DNA level. **c** Schematic illustration of NARS2 at the protein level. Both variants occur at the catalytic domain. Amino acid residues were numbered according to the GenBank reference sequence (NP_078954).

Diagnosis of Leigh syndrome is made based on the neuropathological or neuroradiological findings of bilateral symmetrical lesions within the brainstem and basal ganglia structures. These lesions must be accompanied by elevated levels of lactate in the blood or CSF, indicating abnormal energy metabolism^1^. Lactic acidosis is a hallmark of all mitochondrial diseases, but is neither invariably present nor necessarily severe^14^. Among the previously reported 22 cases with *NARS2*-associated disease, at least seven patients showed normal lactate levels at presentation^4,5,7,10,12,13^. Our case also showed normal lactate levels in both the blood and CSF, but elevated levels in catabolic states such as vomiting, diarrhea, and fever. Recently, several serum biomarkers such as growth differentiation factor 15 and fibroblast growth factor 21 have been reported as diagnostic indicators of mitochondrial diseases; however, a lack of validated biomarkers for diagnosing mitochondrial diseases has not been resolved^15^. A definite diagnosis is only possible with genetic confirmation. Early diagnosis is crucial for optimizing care; avoidance of the drugs (e.g., VPA) metabolized by the mitochondria is recommended. VPA can contribute to mitochondrial dysfunction in proximal renal tubular cells and may cause Fanconi syndrome in patients with mitochondrial diseases^16^. Recognition of these clinical characteristics may facilitate the early diagnosis and proper treatment of patients with Leigh syndrome, improve their long-term outcomes, and help adapt appropriate genetic counseling.

## Supplementary information

Supplementary Figure 1

## Data Availability

The relevant data from this Data Report are hosted at the Human Genome Variation Database at 10.6084/m9.figshare.hgv.3160, 10.6084/m9.figshare.hgv.3163.
