# Supplementary figures and images for: Novel NARS2 variant causing leigh syndrome with normal lactate levels
Source: Hum Genome Var. 2022 May 4;9:12. doi: 10.1038/s41439-022-00191-z (PMC9068749; doi:10.1038/s41439-022-00191-z)

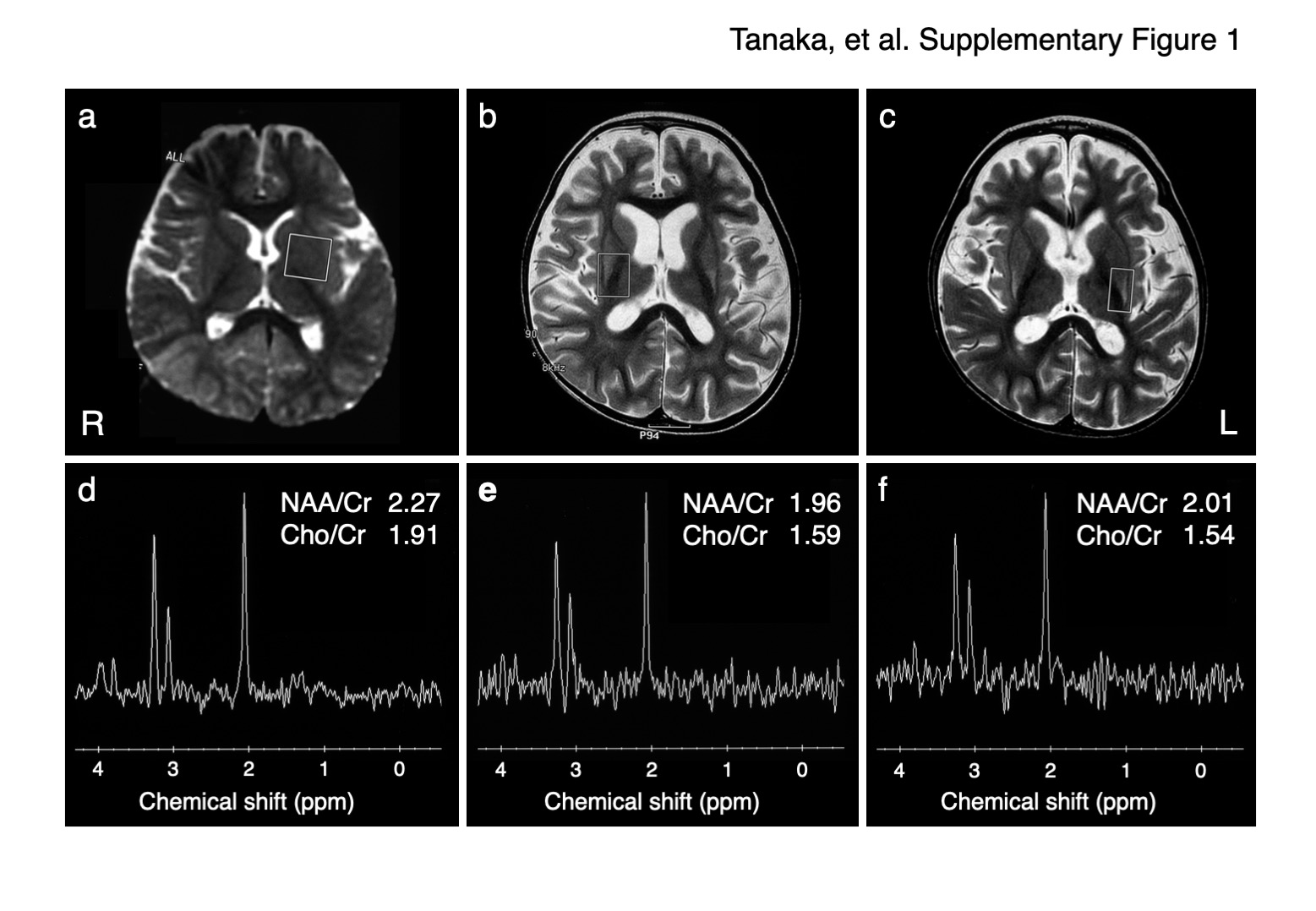

Supplement: Supplementary file 1 — Supplementary Figure 1 [file 41439_2022_191_MOESM1_ESM.jpg]
